# Hindering and Facilitating Factors While Implementing the Family and Community Nursing Model in Italy: Findings from a Qualitative Study

**DOI:** 10.3390/healthcare13091001

**Published:** 2025-04-26

**Authors:** Majda Clodig, Gaia Magro, Paola De Lucia, Marisa Prezza, Gaia Dussi, Sara Dentice, Chiara Moreal, Stefania Chiappinotto, Alvisa Palese

**Affiliations:** 1 Department of Medicine, University of Udine, 33100 Udine, Italy; majda.clodig@uniud.it (M.C.); magro.gaia@spes.uniud.it (G.M.); dussi.gaia@spes.uniud.it (G.D.); dentice.sara@spes.uniud.it (S.D.); moreal.chiara@spes.uniud.it (C.M.); alvisa.palese@uniud.it (A.P.); 2 Regional Coordinating Agency for Health, 33100 Udine, Italy; paola.delucia@arcs.sanita.fvg.it (P.D.L.); marisa.prezza@arcs.sanita.fvg.it (M.P.)

**Keywords:** family and community nurse, consolidated framework for implementation research, hindering, facilitating, experiences, models of care delivery

## Abstract

**Background/Objectives:** Several Italian regions have started to introduce the Family and Community Nurse model. The aim of this study was to describe the facilitating and hindering factors that have influenced the implementation of the model by analyzing regional policies from the perspective of nurses appointed as Family and Community Nurses. **Methods**: A qualitative study from 2023 to 2024 following the Standards for Reporting Qualitative Research. Nurses attending a training course were eligible (N = 68) and multi-method data collection was used. Mandatory written project works were requested at the end of the course, and interviews conducted after six months with 14 purposively selected nurses were used. The data were subjected to content analysis. The factors identified were categorized by level (nurse, microsystem, mesosystem, exosystem, and macrosystem) and by the domains of the Consolidated Framework for Implementation Research. **Results**: A total of 23 facilitating factors and 20 hindering factors were identified across all levels and four domains of the Consolidated Framework for Implementation Research. Implementation required nurses to shift from a task-oriented to a user-oriented approach to care that emphasizes accountability and citizen needs. Facilitating factors were the availability of advanced competencies, digital skills, familiarity with communities, and effective teamwork supported by leaders. Challenges arose from fragmented systems and unmet community expectations. Strengthening cross-sector collaboration, fostering trust, and engaging third sector resources were critical factors hindering holistic, patient-centred care. **Conclusions**: The Family and Community Nurses model promotes integrated, patient-centred care through proactive approaches that require advanced competence and interprofessional collaboration. Training, leadership support, and the removal of systemic barriers are critical. Future research should focus on integrating the identified factors into strategies to optimize the implementation of the Family and Community Nurses model.

## 1. Introduction

The ageing rate of the population has increased in recent decades [1], leading to new challenges, especially for people developing chronic diseases [2], resulting from a combination of genetic, physiological, environmental, and behavioural factors and requiring long-lasting care [3]. The complexity of care required by individuals with chronic diseases has triggered the need to rethink healthcare services [4,5], introduce new models of care delivery, and shift the focus from in-hospital acute settings to people-centred care integrated in the community [1,4]. In this scenario, primary healthcare (PHC) has become the most effective approach to address the needs of individuals with chronic diseases [6]. PHC focuses on the needs of a community, along a continuum from health promotion, prevention, disease detection, and treatment to palliative care [7].

Global and European policies based on the existing studies in the field and on international health recommendations [2,8] have highlighted the need to invest in the Family and Community Nurse (FCN) model as a pivotal method to improve PHC and embrace the complexity of the healthcare demands [9]. However, variations in the implementation of the FCN model have been traced mainly due to peculiarities of the context and healthcare needs of the population across countries and to the levels of autonomy and responsibility given to nurses [10,11]. To enhance inter-country harmonization, the EuropeaN curriculum for fAMily aNd Community nursE (ENhANCE) project has proposed a standardized professional profile for FCNs in Europe based on specific core competencies [12]. Parallel to these scientific efforts, some countries, such as Italy, have started to convert district maintenance to the FCN model since the late 1990s. About ten years later, additional measures to integrate care, promote self-management, and involve community resources were introduced [13] and further refined [14]. In Italy, the competencies expected of FCNs were initially defined by the Italian Chamber of Nurses [15] and, in 2022, by law [16]. FCNs should understand epidemiological data, the main needs of the population, and the network of health and social services in order to take horizontal and vertically integrated actions between services and professionals [17]. In addition, FCNs are expected to identify care needs and demand in primary care, focusing on health promotion and disease prevention. They are also expected to ensure coordination between primary care and other healthcare providers [18]. However, despite increasing clarity on the competencies expected of FCNs and available strategies that also provide harmonization in training, the implementation of the model is still a challenge, with hindering factors preventing its full implementation in practice [11].

### 1.1. Implementation of the FCN Model

The implementation science (IS) framework can be a valuable tool for facilitating the translation of available evidence into primary care practice [19], thereby addressing the significant time lag between evidence generation and its broad application [20]. By identifying and understanding the factors that influence implementation outcomes, the framework supports the adoption and scaling of innovations within complex settings [19]. Specifically, the Consolidated Framework for Implementation Research (CFIR) is particularly well suited for systematically assessing these factors across diverse contexts and new models of care [21,22]. In the field of nursing, Breimaier et al. emphasized the potential of the CFIR to assess baseline, progress, and outcomes achieved during implementation, identify influencing factors through content analysis of qualitative data collected during implementation, and facilitate the interpretation of key findings [23]. In addition, the CFIR framework was used to explore the perceptions and perspectives of stakeholders [24,25]. However, due to the novelty and complexity of the FCN model, the application of CFIR in this context has not yet been sufficiently explored. There is limited research on the implementation of the FCN model. There is some evidence of specific FCN functions, such as the successful approach to diabetes prevention in a Latino community [26] or the improved knowledge of obesity prevention and management in the community [27]. Teamwork has been documented as important, whereas previous working experience has been underlined to promote the implementation of the model [28]. The quality of interactions between the organization and the team and strategies ensuring knowledge sharing, capacity building, and practice integration have also been reported as important [29,30,31]. Above all, specific training has been documented to harmonize the required competencies, the professional vision, and the aims of the role [28,30]. However, despite educational efforts devoted to supporting nurses to become FCNs [32,33], issues regarding competencies and heterogeneity in professional functions [1] are still present, hindering the implementation of the FCN model [34]. Moreover, hindering factors for implementation have been associated with the team and organizational level [32] and the lack of feedback on the effectiveness of the actions taken to implement the model [35]. Therefore, various facilitating or hindering factors play a role, suggesting that a comprehensive strategy is recommended to overcome the challenges encountered [36]. In this context, appreciating the experiences of nurses who have been involved in the implementation of FCN can help leaders and healthcare organizations to identify contextual factors and address the factors preventing the full implementation of the model [37].

### 1.2. Aim of the Study

The aim of this study was to describe the factors that hinder and facilitate the implementation of the FCN model in Italy based on nurses’ experiences. The intention was to fill the gap in the strategies that can facilitate or hinder the transformation of community nursing services in practice in order to inform future policies in different regions and countries.

## 2. Materials and Methods

### 2.1. Study Design

A descriptive qualitative study [38] was performed between June 2023 and April 2024 according to the guidelines of “Standards for Reporting Qualitative Research: A Synthesis of Recommendations” (SRQR) [39] (Table S1).

### 2.2. Setting

This study was conducted in a northeastern region of Italy where there are four hospitals and two research institutes serving more than 1,200,000 citizens [40]. In accordance with the policy established at the regional level [41], FCNs were introduced (Table S2) as “the professional responsible for the care processes in the family and in the community”. According to the standards set by the law [16], a ratio of approximately 1 FCN/3000 people was expected. The policy was guided by global and European recommendations [8,12] and emerging care needs [42].

To promote the implementation of the FCN model, a mandatory structured course was designed for nurses already working in the community. The course included a series of face-to-face lectures on (a) European and Italian healthcare policies, (b) socio-demographic and priority health needs of the Italian and regional population, (c) methods of health promotion and education, (d) the role and responsibilities of nurses, (e) soft and technical evidence-based skills, and (f) strategies to integrate social and health systems. The course lasted 30 h and ended with an assignment: each participant was expected to complete a project as homework that addressed the following insight: “Describe your perception of the FCN model as learned in theory during the course and its implementation in your daily practice”.

### 2.3. Participants and Data Source

Overall, this study included (a) nurses working in the region (b) who participated in the regional training courses for FCNs and (c) were willing to participate in this study.

This study was based on multi-method sources of data:

1. The project work that was mandatorily requested at the end of the course from the nurses who participated in the courses (N = 68): The project works were eligible if they were developed by nurses who participated in the entire course (100% of attendance), completed the form with some professional data, and gave their consent to participate (N = 68); and
2. The experiences of nurses identified in a purposive sample [43] by a researcher (AP) according to the following criteria: Nurses who (i) participated in the course, (ii) submitted meaningful project work, (iii) were still active after six months of the course, and (iv) provided written consent to participate in this study. While all professional data and project work were collected at the end of the course, the purposive sample was identified after six months of implementing the FCN model. None of the invited nurses declined to participate. Purposive sampling was terminated when, in the judgment of the researchers (see authors), data saturation was reached, when the dominant themes were considered closed, and when no further themes emerged from the interviews [44].

### 2.4. Data Collection Process

First, at the end of the course, participants were required to write a project work regarding their current professional experiences to identify (a) the perceived implementation of the FCN model in its key features as learned in the course and (b) strategies to promote its full implementation (Table 1). Nurses’ professional data were also collected using an ad hoc form (Table 1).

Second, a purposive sample of nurses was selected six months after the course. An open-ended audio-recorded interview was conducted asking (a) the level of implementation of the FCN model in practice from 0 (not at all) to 100% (completely) and (b) the factors that hinder or facilitate implementation. An interview guide was developed by the researchers based on the literature available in the field [20,21,22,35,45]. The questions were tested with two nurses to assess comprehensibility and feasibility. No changes were necessary. Interviews were conducted by a researcher (AP) with advanced experience in qualitative research who was involved in FCN policy discussions at the regional level. No relationship was established with the participants before this study began. Participants were only informed about the researcher’s working position and the objectives of this study. The interviews were conducted online via the Zoom platform, and no research members other than the researcher (AP) and the interviewee were present. All interviews were recorded after consent was obtained and lasted between 30 and 60 min. Overall, the data collection on professional characteristics and project work was conducted in June and October 2023; the interviews were conducted between December 2023 and April 2024.

### 2.5. Data Analysis

The professional data were summarized using descriptive statistics (means, standard deviations [SD], frequencies, and percentages). A content analysis framework [46] was then used to summarize both the project work and the individual interviews, which were transcribed verbatim. Three main phases were as follows: preparation, organization and reporting. In the preparation phase, the researchers (MC, SC) read the project works and the verbatim transcribed individual interviews (MC) to gain a general understanding of the data; then, in a second reading, the researchers identified the units of analysis, i.e., sentences or words with a significant meaning according to the aim of this study. In the subsequent organizational phase, an inductive approach was followed: Open coding was conducted, and labels were identified by coding the data obtained. In this phase, data from both project work and individual interviews were considered. Labels were then grouped and assigned to a single theme according to their similarities and differences. A general description of their content as factors gendering or facilitating the implementation was given by providing one or more citations for each theme: quotations from project work were labelled PW (project work) and numbered (1, as the consecutive number of the collected project work); quotations originating from the interviews were instead labelled I (interviews) and then numbered (e.g., 2, as the consecutive number of the interviewee).

Two conceptual frameworks were used to organize the emerging themes and create a meaningful map of factors influencing the implementation process of the FCN model:

(1) The first concerns the levels of factors: The framework developed by Jones et al. [47], which identifies five levels that influence the quality of care provided, was considered. All factors that emerged were located at the appropriate level, namely (i) the level of the nurse as an individual, autonomous agent who makes decisions; (ii) the microsystem level, where care is delivered and where nurses have direct social interactions with patients, colleagues, and direct supervisors; (iii) the mesosystem level, as the set of domains where regular interactions with the community and nurses’ families take place; (iv) the exosystem level, as the set of domains where nurses are not directly involved but where events may affect care (education, legislation, regulatory bodies); and the (v) macrosystem level, where social culture, nurses’ subculture, and all levels of the system interact;
(2) The second concerns the systematic assessment of the complexity of implementation: The CFIR framework [21] was used with its domains of (i) intervention characteristics, examining the characteristics of the intervention itself that may affect its implementation; (ii) outer setting, which focuses on the external context that influences implementation; (iii) inner setting, which refers to the internal context of the organization; (iv) characteristics of individuals, which examines the people involved in implementation; and (v) process descriptions, which include the steps involved in implementing an intervention [21,22]. Each factor was supported with meaningful citations.

### 2.6. Rigour and Truthfulness

Several strategies have been ensured to promote rigour and truthfulness [48,49]. First, credibility was promoted by selecting experienced participants and using direct quotations from interviews and project works. Second, transferability was promoted by describing the setting, participants’ profiles, and the data analysis process. Third, dependability was ensured through a collaborative approach between researchers in the data analysis process, while confirmability was ensured by combining different data collection methods (project works and interviews).

## 3. Results

### 3.1. Participants and Data Source

A total of 68 project works were analyzed, comprising 320 pages (minimum 2 pages and maximum 8 pages, average 4.7 pages per paper). They were developed by nurses who participated in the course (Table S3). They were predominantly women (62/68) with a mean age of 46.9 years (SD 7.5). Most of them had a diploma in nursing (42/68), and 21/68 had also completed postgraduate training, mostly in management (6/21) and community nursing (4/21). Their total experience averaged 9.2 years (SD 8.2), mainly in hospitals (41/68). They worked in the community for an average of 13.9 years (SD 7.2).

A total of 14 nurses who participated in the course and provided their project work were interviewed (Table 2). All were female and had an average age of 51.3 years (SD 7.6). Most of them had a diploma in nursing (12/14), and about half of them (7/14) had also completed postgraduate education. Overall, they had an average of 30.7 years of experience (SD 8.5), mainly in a hospital (8/14) or in both hospital and community settings (4/14). At the time of the survey, the nurses had been working in the community for an average of 15.3 years (SD 6.2), mostly as FCNs (11/14). According to their assessment, the degree of implementation of the FCN model was between 30% and 80% (mean 58.3%; SD 14.7%).

### 3.2. Levels of Facilitating and Hindering Factors

A total of 43 factors were identified, 23 of which facilitated the implementation of the FCN and 20 of which hindered it. Specifically, these were factors related to the nurse level (nine facilitating and six hindering factors), the microsystem (four and four, respectively), the mesosystem (four and three), the exosystem (two and two), and the macrosystem (four and five) (Table S4).

#### 3.2.1. Nurses

Participants faced a change in their approach to care when they were asked to start implementing the FCN model. The presence of nurses who tended to be more patient-centred facilitated implementation, while nurses who focused on their own perspective or performance and tasks faced challenges in this process. The shift from a performance-oriented, task-based model to a model focused on responsibilities and processes was perceived to interfere with implementation. These changes in professional perspective have facilitated the transition to a more complex, structured, and tailored organizational model focused on user needs. The feeling of having time to care for patients has also had an impact on implementation. The avoidance of any form of hurry during care has helped to promote the full implementation of the FCN model, while the amount of bureaucratic and logistical activities that require time, as well as the perception of being overwhelmed by the tasks to be completed, have hindered the process. The existence of advanced competencies and access to specialized nurses, according to the complexity of the care required, have facilitated the implementation of the FCN model. On the other hand, the lack of ability to prioritize or the lack of availability of specialized nurses has delayed the process. As they were not equipped with such advanced competencies, the availability of specialized nurses was essential to receive appropriate guidance when needed. Moreover, digital skills facilitated the implementation of the FCN model in ensuring complex functions such as remote monitoring, education, and prevention consultations.

#### 3.2.2. Microsystem

Facilitating factors related to the proximity of nurses to people, their families, and communities, enabling them to understand the needs, develop effective partnerships, know the available networks, and understand the development trajectories of the same community. On the other hand, unfamiliarity with all these elements prevented the full implementation of the FCN model. First, the nurses reported that being close to the users, their families, and a specific community, without rotating in other communities, facilitated a deep understanding of the needs, the development of a strong partnership (with the families, the networks), and the recognition of the nurses as a reference point. The stability and resulting familiarity with the context facilitated the implementation of the FCN model. In addition to the proximity to the people cared for and their families, the deepening of the formal and informal networks available facilitated the provision of holistic care and the continuity of support to families when nurses were not available. On the other hand, the implementation of the FCN model was hindered when needs were not identified in advance or were not fully prioritized by nurses; the implementation of the FCN model was hindered because caregivers provided care that did not meet the family’s expectations. In these cases, the level of involvement of families was also limited because they needed time to understand the new model, as they were expected to move from being simple recipients of tasks at home to full partners in the care processes. Therefore, the nurses reported that the introduction of the FCN model had a positive impact on both the users and their families and increased their confidence in the model. Nurses should also be present in the community on a daily basis. This helps them to understand the dynamics and development of the community, including the social, cultural, and economic aspects. For example, if the nurses were not familiar with the community, the relationship with minority groups, with language barriers, was hindered. Thus, if nurses were able to deepen their familiarity with the community, the implementation of the FCN model was facilitated. On the other hand, when such deepening was not possible, such as with the constant rotation of nurses, the implementation of the FCN model was hindered because the nurses did not go beyond simply performing the required tasks.

#### 3.2.3. Mesosystem

At the level of the mesosystem, several factors have emerged that all describe the professional and working environment of nurses. Working as a team and sharing information to tailor care was cited as important; the availability of shared protocols and interoperable and integrated electronic documentation, all of which ensure accessibility of data and timely availability, was seen as valuable. Moreover, regular case discussions, data sharing and tailored care were considered important. Lack of collaboration or communication with other professionals (e.g., General Practitioners [GPs] and social workers) led to significant gaps in care. Similarly, delays caused by unanswered emails and phone calls resulted in wasted time and hindered the effective implementation of the FCN model. In addition to the role played by the team, support from nursing leadership was also seen as critical, as they were able to encourage reflection on the day-to-day implementation of the model and address organizational barriers. Without guidance from managers, nurses felt isolated, which threatened their self-confidence in implementing the FCN model. In addition, a positive work climate facilitated implementation by fostering cohesion and strengthening the ability to build effective and sustainable professional networks. Such an atmosphere enabled information sharing and motivated nurses to adopt new practices. However, implementing the FCN model without the necessary human resources and stability of nurses has been noted as a challenge.

#### 3.2.4. Exosystem

Nurses reported two main factors in relation to the role of GPs and institutions. While both were involved in the process of moving from the previous model to the FCN-based model, nurses felt legitimized and fully supported in their new role. GPs have a recognized role in society; they are the first point of contact for patients, families, and communities. Given their recognized role, their involvement played a crucial role in promoting continuity of care and strengthening ties with the community. Therefore, their early involvement in change facilitated the implementation of the FCN model. In contrast, when nurses worked within immanence systems where there were tensions between services and professionals, the implementation of the model was hindered. In addition, some institutions (e.g., the mayors of some cities) lobbied strongly for formal recognition of the FCN model and demanded the implementation of the model when it was not available. However, in other municipalities, nurses reported a lack of expected support due to feeling blocked or being described negatively by newspapers, which prevented the full implementation of the model.

#### 3.2.5. Macrosystem

The gradually growing awareness of the possibilities of the FCN model was described as positive, suggesting that information and the education of all citizens are important. On the other hand, the unwillingness of many families to engage with some interventions hindered the implementation of the FCN model. Nurses described it as difficult to be accepted as an FCN because families showed little or no interest in preventive interventions. This resistance often resulted from a lack of need for such models of care, which limited acceptance and engagement. In addition, the discrepancy between community expectations and the actual level of services provided was cited as a major barrier to healthcare. Such discrepancies can arise when users, families, or communities have different perceptions and expectations of care services, as was the case with the different interpretations of the role of FCNs, leading to misunderstandings and dissatisfaction. However, effective communication and transparent education about the services provided are able to bridge gaps and promote trust and mutual understanding. Building networks within the community has also been identified as important. To overcome this hurdle, continuity of care is essential, as a consistent and reliable presence makes professionals anchors in the community. Over time, this trust enables people to seek advice and care earlier, which supports proactive health management. Nurses reported that the fragmentation of service systems hindered the FCN model; problems in seamless communication between healthcare departments and external organizations led to inefficiencies, while integrated systems based on patient-centred networks encouraged implementation. Collaboration between departments and external organizations strengthened the implementation processes. The lack of links with the third sector, such as voluntary associations, and the underutilization of nurses’ full potential were cited as threats to the FCN model. It was recommended that third sector resources should be proactively identified and included in the redesign of health services. An open-minded approach to engaging these organizations facilitated the implementation of the FCN model in all its functions.

### 3.3. Evaluation of the Complexities of the FCN Implementation

**Figure 1:**
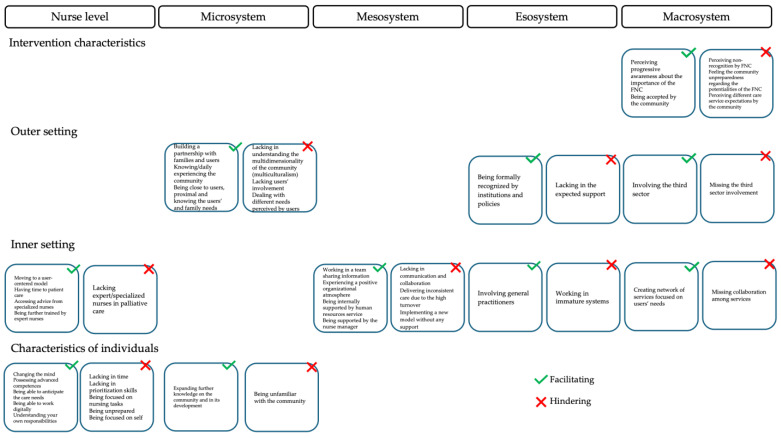
Facilitating and hindering factors according to the Consolidated Framework for Implementation Research [21,22].

The 43 factors categorized in the five levels were then organized into the CFIR domains, namely intervention characteristics, outer setting, inner setting, and characteristics of individuals, as shown in Figure 1.

#### 3.3.1. Intervention Characteristics

Two facilitating and three hindering factors were identified, all of which are located at the macro-level (Figure 1). A key hindrance was the lack of recognition of the role of FCNs by the community (“*In my experience, the role of the FCN is not always recognised*” I5). The complexity of the model further complicated its implementation, as it required the involvement of multiple stakeholders with different perceptions (“*Discrepancy between the expectations of users/family members and the actual perceived situation*” PW7). Furthermore, complexity increased with the number of people affected, while communities remained oriented towards a hospital-centred model and underestimated both prevention and community-based services (“*As for families (…) they don’t see the problem and don’t see the need and don’t want to work at the level of prevention*” I3). Strategies for adapting the FCN model to local circumstances and needs were highlighted to promote its implementation: Nurses reported a greater awareness of the role of the FCN when citizens are involved (“*Their attitude, with the involvement of the nurse explaining to the families themselves what the role of the community nurse is, that should make the difference, or at least start to make the difference*” I12).

#### 3.3.2. Outer Setting

Five hindering and five facilitating factors emerged, involving the micro-, meso-, and macro-levels (Figure 1). The participants emphasized that the FCN model effectively addresses the health needs of older adults with chronic diseases. After training and implementation, nurses began to build partnerships with patients and families, recognizing their importance in providing quality care (“*Closeness, so familiarity, you know, a more empathic way of relating to people*” I5). Such connections also clarified the role and responsibilities of the FCN across the communities, where different expectations were expressed (“*There are also different expectations, you know, then of the district nurse… different levels on interpretation and consideration of our service*” I12). External policy support facilitated the implementation of the FCN model as it aligned with national and regional strategies to address healthcare challenges (“*The institutions, the local authorities are willing to do this… maybe it’s a source of pride for the local authorities to have a nurse in the community*” I9). However, limited openness to the world hindered progress. Health systems lacked links with external organizations (“*There are unfortunately no links with the third sector sadly (…) it would be good if we created links, pathways, roads that would facilitate the situation of users in this case*” I7). Nevertheless, the involvement of the third sector was also seen as beneficial (“*Basically we are paying a bit more attention to the third sector now*” I1).

#### 3.3.3. Inner Setting

Ten facilitating and six hindering factors were identified at all levels, except for the microsystem (Figure 1). One important factor was the shift towards a culture that puts the patient at the centre and emphasizes holistic care over task-oriented approaches (“*It is about moving from an activity that is not just performance-oriented to a much more complex, structured and personalized organizational model that focuses on the needs” of the user*” I14). Support and mentoring from experienced colleagues were crucial in developing skills and solving problems (“*I am a long-time member of the group, and I support the two new colleagues in my field by giving them information and strategies to solve specific problems*” I2). The establishment of professional networks and specialized roles also improved care (“*We now have a nurse who specifically does these treatments, so we are doing this implementation in some areas to be more specialized*” I8). Team building and effective communication between healthcare professionals were highlighted by nurses as essential to promoting collaboration and implementing the model (“*Working in a team requires a relationship between people; physical proximity between the different professionals fosters the relationship. Direct vision and eye contact create an important communicative link*” PW21). A positive organizational climate facilitated interpersonal communication and relationship building (“*Positive atmosphere that enables the transfer of information within the group, so the ability to pass on the information to everyone, to motivate them also in terms of change (…) to pass on information, you have to build good relationships, and sometimes the atmosphere influences the relationship*” I13). Organizational incentives, the availability of resources, and leadership were key factors that made this possible. Leadership played a crucial role in encouraging innovation and shared responsibility (“*The nursing management also encourages us and insists on this concept; we talk about it, so we tried to see, to do, what do you say, when one decided to try, the others usually followed her*” I1). Adequate human resources were considered essential (“*The fundamental element is the need to channel and organize the human resources available to improve the quality of the responses offered by the service*” I14). Conversely, fragmented communication and collaboration between hospital and community hindered progress (“*I noticed that years ago, before COVID, we were better able to track people in their complexity (…) they called me, they communicated with us, which is no longer the case today (…)*” I7). Overall, the implementation process was still at an early stage and required further time and effort to fully mature (“*I have the impression that we are not yet mature and ready enough, both in terms of people and the services around us, to fully implement this model*” I2).

#### 3.3.4. Characteristics of Individuals

Six facilitating and six hindering factors were associated with the characteristics of the individual domain, and these related to the nurse and microsystem levels (Figure 1). Participants reported challenges in prioritizing time and skills (“*Nurses are overloaded with many things*”, I11) and low self-efficacy in implementing the FCN model (“*Because in my opinion we focus on our own, what do you call it, just our own step, you know, so we lack the overview*”, I13). After attending the course, nurses reported increased self-confidence, higher levels of self-efficacy (“In *the beginning we were more performance-oriented, now we have become so, we have started to work more in terms of patient care*”, I3), and an increasing awareness of the knowledge and skills required to manage the complexity of change. As a result, nurses began to recognize the importance of transforming the nursing model and focused on developing and enhancing new competencies and skills.

## 4. Discussion

### 4.1. The Study

As far as we know, this is the first Italian study to describe the implementation of the FCN model of care in practice. Previous studies have attempted to describe different models of care [50,51] that differ in their basic principles, community needs, and approaches. Overall, our findings reflect the actualization of a policy in terms of general or widespread implementation, not just a pilot project [50]. As these were nurses who were already working in the community, the change was mainly based on revising the nursing service from a task-oriented service to one based on the FCN model. Examining the implementation process experienced by the nurses’ sheds light on how to facilitate the complex transition from a task-oriented model (home care) [52] to a process-oriented model (FCN model) [53,54]. We involved experienced nurses who had the responsibility to change their practice after learning the key principles of the FCN model in a face-to-face course and developing a project work as an assignment that encouraged reflection on action [55]. The key professional characteristics of the participants are consistent with those documented for nurses working in community settings in Italy [56]. They reported varying degrees of implementation of the FCN model, ranging from 30% to 80%, suggesting that they were still experiencing the professional transition from the previous model to the new model after six months of training. Further analysis of the implementation process through longitudinal studies could help to adjust the necessary strategies over time.

### 4.2. Factors Facilitating and Hindering the FCN Implementation

A comprehensive map of factors emerged, 43 in number, which almost balanced each other out when considering the promoting and inhibiting factors. Several factors have worked in both directions, as negative and positive forces, and sometimes represent two sides of the same coin, as facilitating or hindering factors. The overall map shows that strategies are needed at different levels, from the nurse level to the macrosystem. By categorizing the CFIR domains, additional elements of this strategy have emerged, in which the outer and inner setting, the degree of innovation implemented, and the individuals [21,22] should also be considered. No factors have emerged in the process domain of the CIFR, which comprises the steps for implementing an intervention, as the data collection was carried out during the first phase of implementing the FCN model.

Overall, communities are not prepared for a new model based on patient-centred care, which could prevent FCN recognition. In the same implementation processes, nurses report increasing awareness among users, families, and the community, in part because the FCN model can provide a viable solution to their needs. Nurses emphasize that awareness and acceptance of their role in the community have changed. Collaborative planning, building relationships among stakeholders, and adapting the model to the community can increase the likelihood of success [24,57]. Most hindering and facilitating factors reflect the complexity of the FCN model with its various components (e.g., family and community involvement, collaboration between services, multidisciplinary approach) that may prevent a full understanding of its potential. In addition, health systems should focus on care coordination [58]: The FCN model should therefore be seen as holistic and reflect a transformation of the whole health system, not just a single part. FCN requires a set of assumptions that are external to the nursing service and are an expression of the whole system in which relationships, roles, decision-making processes, and the same care process should be transformed. Therefore, informative and educational interventions are recommended to improve citizens’ knowledge. In addition, insufficient collaboration and coordination between services, especially between hospital and primary care, should be overcome with the help of networks and care pathways.

Within the outer setting domain, the fact that the FCN model has been established through a policy and subsequent training can encourage innovation. However, in challenging times, maintaining a negative narrative about change and difficulties in healthcare requires additional strategies. Replacing negative narratives with positive ones can uplift, motivate, and empower professionals, benefiting the healthcare system as a whole [59]. In addition, links with the third sector, traditionally an important part of the health system, can facilitate the implementation process [60]. However, even in this case, partnership requires an overall strategy to achieve the potential benefits: working with the third sector is challenging due to differences in local practices that may require specific additional interventions [61].

In terms of the inner setting domain, the FCN model is designed as a person-centred model, considering the needs and preferences of patients, families, and communities. The implementation of the FCN model is also associated with a change in the perspective of nurses. The transition from a task-oriented to a user-centred model requires an awareness of responsibility and competence. Refocusing professional paradigms on patients and families requires a cultural shift and appropriate resource allocation and professional development opportunities. High turnover rates or a high administrative burden may interfere with the implementation of the FCN model [24]. In addition, the implementation of the model may be affected by some critical decisions, such as identifying (or not identifying) FCNs among nurses living in the community and applying (or not applying) professional rotations to ensure regular replacement of FCNs and avoid the risk of being overwhelmed by the emerging needs of the community. Hospital rotations have been studied to promote enthusiasm and creativity, strengthen communication and collaboration between departments, and improve some competencies [62]. Rotations can help nurses to cope with overwhelming situations [63]. On the other hand, rotations can also prevent an understanding of developments in the community, such as the emerging needs of minority groups [64]. Overall, while local recruitment of nurses is a mandatory decision in some contexts (in rural areas) to provide nurses required care, no recommendations for the role of the FCN have been developed in practice. While proximity to people and communities promotes partnerships and an understanding of local needs, the lack of effective communication between team members is a significant barrier. Strengthening interprofessional communication is critical for the sustainable implementation of the model. This has also been emphasized in previous studies [24], where the role of GPs has been underlined, which was also reflected in our results [65]. The availability of integrated electronic documentation systems and streamlined work processes can facilitate communication and collaboration between team members, mitigate inefficiencies, provide structured workflows [66], and measure outcomes [67], all of which support the implementation of the FCN model.

In the individual domain, nurses feel that they play a key role in changing their nursing perspective, improving time management, and acquiring new skills, which require education [24,30,68,69]. However, the professional paradigm shift that the new model seeks to achieve requires additional strategies, including mentoring: the provision of continuing education and the identification of specialized nurses to provide support in complex situations are critical resources that the system must provide to facilitate the transition. At the mesosystem level, the involvement of senior and mid-level managers and their support system can also facilitate successful implementation. Their influence within professional networks can encourage the adoption of the FCN model and reduce resistance to change. At the macrosystem level, our findings emphasize the importance of early engagement and co-design by nurses to promote adoption and align the model with community needs. Early engagement of caregivers, the community, and those likely to be affected by innovation has been shown to be important in gaining support and increasing the likelihood of the model’s success [10,61].

### 4.3. Limitations

We have included nurses who are already working in the community and have previous hospital experience; their transition to the new model may follow different patterns than those of nurses who have just graduated or have no previous experience in the community. However, their profile, mainly female (see nurses producing the project work) and senior nurses, reflects the main characteristics of Italian nurses working in the community [12,17]. Moreover, the duration of experience in the FCN model was not a selection criterion, and this aspect might have influenced the nurses’ own perception of the model. Furthermore, the hindering factors were not weighted to analyze whether they were able to override the facilitating factors in a dynamic that could strengthen or hinder implementation. In addition, this study was conducted in northern Italy, and the regionalization of healthcare could pose different challenges to FCN implementation in other regions [70]. All these factors suggest that future studies should include diverse participants in terms of gender, experience as a nurse, regions, and experience in the FCN role.

## 5. Conclusions

This study describes the barriers and facilitators to implementing the Family and Community Nursing (FCN) model in Italy based on the experiences of nurses directly involved in the process. The implementation of the FCN model has both challenges and facilitating factors that influence its full integration into practice. Early identification of these factors at the local level can help in developing targeted strategies to overcome existing barriers. In addition, the accumulation of evidence with more research can inform the development of strategies at the national and international levels to support wider adoption of the FCN model. Both strategies and policies should consider the multifaceted challenges, not only those at the individual nurse level, e.g., by delivering a training course, but also at the organizational and systemic levels. Furthermore, the strategies should cover different domains and consider the specific characteristics of the model and the context in which it is applied.

Overall, the findings suggest that continuing education and advanced nursing competencies are crucial for promoting a patient-centred, process-oriented approach. Other important factors include interprofessional collaboration, the integration of digital communication tools, and the ability to overcome systemic barriers. Healthcare leaders are therefore called upon to identify supporting elements while actively working to break down barriers to implementation. A multi-level, systems-based approach combined with iterative strategic adjustments is essential to align the model with the needs of patients, families, and communities. In addition, community-based information campaigns that build public trust in the model can help raise realistic expectations and encourage active participation in care.

Future research should focus on translating these factors into actionable strategies that can optimize the implementation process. The map of identified factors can also serve as a useful reference in the development of strategies to implement the FCN model in Italy and other European countries, with the aim of comparing strategies in a complex implementation landscape and promoting cross-border harmonization to improve health outcomes. Finally, the involvement of nurses in the FCN model implementation process provides valuable insights into its complexity; their voices need to be heard as this transformation directly affects their professional roles and responsibilities.

## Figures and Tables

**Table 1:** Professional form for data collection and project work structure.

**Professional Data Collection Form**
Age Gender Nursing education attended Experience in community setting (years) Prevalent functions in the last year (in light of the FCN model) Overall working experience (years, setting, professional role)
**Project work structure**
Please, describe your current work experience in the community field, comparing your daily practice with the FCN model established by regional policy Please describe the similarities/differences of your daily practice with the FCN model and strategies that are facilitating the model implementation

Legend: FCN: Family and Community Nurse.

**Table 2:** Characteristics of participants involved in the individual interviews (n = 14).

ID	Gender	Age, Range of Years	Undergraduate Education	Postgraduate Education	Working Experience, Years	Prevalent Field	Experience in the Community Field, Years	Role in Community Field	FCN Model Implementation (%)
1	F	50–55	ND	Bioethics	30–34	Hospital	20–24	FCN	50
2	F	40–44	BNS	Wound care and management	20–24	Hospital and community	15–19	FCN	60
3	F	30–35	BNS	End-of-life	5–9	Hospital and community	5–9	FCN	60
4	F	50–55	ND	-	30–34	Hospital	5–9	FCN	80
5	F	45–49	ND	-	20–24	Hospital	5–9	FCN	50
6	F	45–49	ND	-	25–29	NR	20–24	FCN	50
7	F	56–60	ND	-	35–39	Hospital and community	15–19	FCN	70
8	F	50–55	ND	Management and MNS	30–34	Hospital	20–24	Nurse manager	70
9	F	56–60	ND	-	35–39	Hospital	15–19	FCN	80
10	F	45–49	ND	-	30–34	Hospital and community	20–24	FCN	70–80
11	F	50–55	ND	Management	35–39	Hospital	15–19	Nurse manager	50
12	F	56–60	ND	-	30–34	Hospital	15–19	FCN	50
13	F	56–60	ND	Management	40–44	Community	15–19	Nurse manager	50–60
14	F	45–49	ND	Emergency	35–39	Hospital	5–9	FCN	30

Legend: F: female; M: male; BNS: Bachelor of Nursing Science; MNS: Master of Nursing Science; ND: nursing diploma; NR: not reported; FCN model implementation from 0% (not implemented at all) to 100% (totally implemented).

## Data Availability

The data that support the findings of this study are available from the corresponding author upon reasonable request.

## Supplementary Materials

The following supporting information can be downloaded at https://www.mdpi.com/article/10.3390/healthcare13091001/s1, Table S1. Standards for Reporting Qualitative Research (SRQR); Table S2. Brief description of the competencies and functions of Family and Community Nurse; Table S3. Nurses attending the training course and providing their project work (N = 68); Table S4. Facilitating and hindering factors emerged from project work and interviews: levels, themes, and quotes.

## Abbreviations

The following abbreviations are used in this manuscript:

PHC	Primary healthcare
FCN	Family and community nurse
ENhANCE	EuropeaN curriculum for fAMily aNd Community nurse
IS	Implementation science
I	Interview
SRQR	Standards for Reporting Qualitative Research: A Synthesis of Recommendations
SD	Standard deviation
PW	Project work
CFIR	Consolidated Framework for Implementation Research
