# Supplementary material for: Hindering and Facilitating Factors While Implementing the Family and Community Nursing Model in Italy: Findings from a Qualitative Study
Source: Healthcare (Basel). 2025 Apr 26;13(9):1001. doi: 10.3390/healthcare13091001 (PMC12071618; doi:10.3390/healthcare13091001)
Supplement: Supplementary file 1 [file healthcare-13-01001-s001.zip › healthcare-3546835-supplementary.pdf]

Table S1. Standards for Reporting Qualitative Research (SRQR) (O'Brien, B. C.; Harris, I. B.; Beckman, T. J.; Reed, D. A.; Cook, D. A. Standards for Reporting Qualitative Research: A Synthesis of Recommendations. Academic Medicine, 2014, 89 (9), 1245–1251. https://doi.org/10.1097/ACM.0000000000000388)

	Section and Criteria	Description of the Criteria	Section
**Title and Abstract**			
S1	Title	Concise description of the nature and topic of the study. Identifying the study as qualitative or indicating the approach (e.g. ethnograpy, grounded theory) or data collection methods (e.g. interview, focus group) is recommended.	Title
S2	Abstract	Summary the key elements of the study using the abstract formato of the intended pubblication; typically includes background, purpose, methods, results and conclusion	Abstract
**Introduction**			
S3	Problem formulation	Description and significance of the problem/phenomenon studied; review of relevant theory and empirical work; problem statement	Introduction
S4	Purpose or research question	Purpose of the study and specific objectives or questions	Aim of the study
**Methods**			
S5	Qualitative approach and research paradigm	Multi-method qualitative approach (project work, semistructured questionnaires individual open-ended interview), used a content analisys framework<br><br>Qualitative approach (phenomenology) and guiding theory if appropriate; identifying the research paradigm (e.g., postpositivist, constructivist/interpretivist) is also recommended; rationale**	Material and Methods<br>Study Design
S6	Researcher characteristics and<br>reflexivity	Researcher personal attributes, qualifications/experience and relationship with participants'<br><br>Researchers' characteristics that may influence the research, including personal attributes, qualifications/experience, relationship with participants, assumptions, and/or presuppositions; potential or actual interaction between researchers' characteristics and the research questions, approach, methods, results, and/or transferability.	Material and Methods<br>Data collection process
S7	Context	North-Est healthcare trust<br>Setting/site and salient contextual factors; rationale**	Material and Methods<br>Setting
S8	Sampling strategy	How and why research participants, documents, or events were selected; criteria for deciding when no further sampling was necessary (e.g., sampling saturation); rationale**	Material and Methods<br>Participants and data source<br>Data collection process
S9	Ethical issues pertaining to human subjects	Documentation of approval by an appropriate ethics review board and participant consent, or explanation for lack thereof; other confidentiality and data security issues	Material and Methods<br>Institutional Review Board Statement
S10	Data collection methods	Types of data collected; details of data collection procedures including (as appropriate) start and stop dates of data collection and analysis, iterative process, triangulation of sources/methods, and modification of procedures in response to evolving study findings; rationale**	Material and Methods<br>Data collection process<br>Table 1
S11	Data collection instruments and technologies	Description of instruments (e.g., interview guides, questionnaires) and devices (e.g., audio recorders) used for data collection, if/how the instrument(s) changed over the course of the study	Material and Methods<br>Participants and Data Source
S12	Units of study	Number and relevant characteristics of participants, documents, or events included in the study; level of participation (could be reported in results)	Material and Methods<br>Participants and Data Source
S13	Data processing	Methods for processing data prior to and during analysis, including transcription, data entry, data management and security, verification of data integrity, data coding, and anonymization/deidentification of excerpts	Material and Methods<br>Data analysis
S14	Data analysis	Process by which inferences, themes, etc., were identified and developed, including the researchers involved in data analysis; usually references a specific paradigm or approach; rationale	Material and Methods<br>Data analysis
S15	Techniques to enhance trustworthiness	Techniques to enhance trustworthiness and credibility of data analysis (e.g., member checking, audit trail, triangulation); rationale	Material and Methods<br>Rigour and trustfulness
**Results/findings**			
S16	Synthesis and interpretation	Main findings (e.g., interpretations, inferences, and themes); might include development of a theory or model, or integration with prior research or theory	Results<br>Table 2<br>Table S3<br>Table S4
S17	Links to empirical data	Evidence (e.g., quotes, field notes, text excerpts, photographs) to substantiate analytic findings	Results<br>Table S4
**Discussion**			
S18	Integration with prior work, implications, transferability, and contribution(s) to the field	Short summary of main findings; explanation of how findings and conclusions connect to, support, elaborate on, or challenge<br>conclusions of earlier scholarship; discussion of scope of application/generalizability; identification of unique contribution(s) to scholarship in a discipline or field	Discussion<br>Table S4

S19	Limitations	Trustworthiness and limitations of findings	Discussion<br>Limitations
**Other**			
S20	Conflicts of interest	Potential sources of influence or perceived influence on study conduct and conclusions; how these were managed	None
S21	Fundings	Sources of funding and other support; role of funders in data collection, interpretation, and reporting	None

Table S2. Brief description of the competencies and functions of the Family and Community Nurse (Ministero della Salute. Regolamento Recante La Definizione Di Modelli e Standard per Lo Sviluppo Dell'assistenza Territoriale Del Servizio Sanitario Nazionale; 2022).

Collaborates in the anticipation of health needs and carries out health promotion, prevention and management in all age groups
Contributes to the planning of activities, including through care management tools aimed at keeping the population healthy by addressing the needs of individuals, both in terms of prevention and treatment of chronic diseases;
Promotes accessibility and orientation to services to ensure effective care for the person;
Promotes the active and informed participation of the community by organizing group health education activities, either in person or online, in collaboration with all health levels actors, in accordance with the indications of the Department of Prevention and Public Health;
Promotes information/communication activities for both individuals and the community, using appropriate relational skills and languages, formats and interaction modes according to the needs of the target population;
Carries out nursing counselling activities and contributes to motivational support to promote effective behavior encouraging individual and collective participation and empowerment;
Values and promotes the active involvement of the person and his/her caregiver;
Works in strong integration with health- and social- networks and community resources (e.g. associations, volunteers) and works as a team with general practitioners, primary care pediatricians and other healthcare professionals;
Systematically uses digital (e.g. electronic health records) telemedicine and telecare tools (e.g. telemonitoring, teleconsultation, telereferral).

Table S3. Nurses attending the training course and providing their project work (N=68)

ID	Gender	Age, Range of Years	Undergraduate education	Postgraduate education	Working experience in other fields, years and fields	In the community field, range of years	Prevalent function
1	M	40-44	BNS	Management	7, Hospital and community	5-9	Home care nurse
2	F	36-39	BNS	Management	7, Hospital	5-9	Community Nurse
3	M	30-35	BNS	Community nursing	5, Hospital and community	5-9	Case manager
4	F	50-55	Nursing Diploma	-	3, Hospital and community	30-34	Case manager
5	F	40-44	BNS	Palliative care	0.5, Hospital	5-9	Home care nurse
6	F	50-55	Nursing Diploma	-	14, Hospital	15-19	Nurse manager
7	F	45-49	Nursing Diploma	-	5, Hospital	20-24	Case manager
8	F	56-60	Nursing Diploma	-	NR, Hospital	15-19	Case and care manager
9	M	36-39	BNS	Coordination and community nursing	7, Hospital	5-9	Community Nurse
10	F	36-39	BNS	Type not specified	3, Hospital	10-14	Home care nurse
11	F	40-44	BNS	-	2, Hospital and community	15-19	Community Nurse
12	F	45-49	Nursing Diploma	-	19, Hospital	5-9	Home care nurse
13	F	36-39	BNS	Community nursing	0.5, Hospital and community	10-14	Case manager
14	F	50-55	Nursing Diploma	-	10, Hospital	15-19	Case, care, and disease manager
15	F	56-60	Nursing Diploma	-	31, Hospital	5-9	Home care nurse
16	F	50-55	Nursing Diploma	Bone marrow transplant nurses	26, Hospital	5-9	Community Nurse
17	F	40-44	BNS	-	NR, NR	15-19	NR
18	F	45-49	Nursing Diploma	Wound care	6, Hospital	15-19	Case and care manager
19	F	30-35	BNS	-	NR, NR	5-9	Home care nurse
20	F	40-44	BNS	Wound care	3, Hospital	15-19	Home care nurse
21	F	40-44	Nursing Diploma	Coordination, end-life and palliative care	11, Hospital	5-9	Case manager
22	F	40-44	BNS	-	15, Hospital and community	5-9	Community Nurse
23	F	45-49	Nursing Diploma	-	7, Hospital	20-24	Case manager
24	F	45-49	Nursing Diploma	-	1, Hospital	20-24	Case manager
25	F	45-49	Nursing Diploma	-	3, Hospital and community	15-19	Case manager
26	F	56-60	Nursing Diploma	-	5, Hospital and community	30-34	Home care nurse
27	F	50-55	BNS	Coordination	7, Hospital	15-19	Home care nurse
28	F	45-49	Nursing Diploma	-	2, Hospital	15-19	Home care nurse
29	F	45-40	BNS	Coordination	NR, NR	5-9	Case and care manager
30	M	56-60	Nursing Diploma	-	7, Hospital	25-29	Case manager
31	F	30-35	BNS	-	1, Hospital	10-14	Case manager
32	F	36-39	BNS	Coordination	NR, NR	10-14	Case manager

33	F	40-44	BNS	Forensic nursing	2, Community	10-14	Case manager
34	F	56-60	Nursing Diploma	-	14, Hospital	NR	Home care nurse
35	F	50-55	Nursing Diploma	-	4, Community	20-24	Case and care manager
36	F	30-35	BNS	-	NR, NR	5-9	Case manager
37	F	50-55	Nursing Diploma	-	24, Hospital	10-14	Home care nurse
38	F	50-55	Nursing Diploma	MNS	10, Hospital	10-14	NR
39	F	50-55	Nursing Diploma	-	4, Hospital	5-9	Home care nurse
40	F	36-39	BNS	-	8, Hospital	5-9	Case manager
41	F	61-65	Nursing Diploma	-	4, NR	25-29	Case and care manager
42	F	30-35	BNS	-	1, Hospital	5-9	Home care nurse
43	F	56-60	Nursing Diploma	Wound care	1, Hospital	25-29	Home care nurse
44	F	45-49	Nursing Diploma	-	1, Hospital	20-24	Care manager
45	M	36-39	BNS	-	6, Hospital	5-9	Case and care manager
46	F	40-44	BNS	-	NR, NR	10-14	Case and care manager
47	F	50-55	BNS	Forensic nursing	5, Hospital	20-24	Case and care manager
48	F	45-49	Nursing Diploma	-	6, Hospital	15-19	Case and care manager
49	F	40-44	BNS	Palliative care	11, Hospital and community	5-9	Case manager
50	F	45-49	Nursing Diploma	Integrated care	20, Community	5-9	Home care nurse
51	F	40-44	BNS	-	15, Hospital	5-9	Community Nurse
52	F	45-49	Nursing Diploma	-	7, Hospital	15-19	Community Nurse
53	F	56-60	Nursing Diploma	-	20, Hospital	15-19	Case manager
54	F	56-60	Nursing Diploma	-	30, Hospital	5-9	Case and care manager
55	F	50-55	Nursing Diploma	-	NR, NR	15-19	Case manager
56	F	45-49	Nursing Diploma	-	7, Hospital	20-24	Case and care manager
57	F	45-49	Nursing Diploma	-	NR, NR	15-19	Case and care manager
58	F	56-60	Nursing Diploma	-	18, Hospital	5-9	NR
59	F	50-55	Nursing Diploma	-	9, Hospital	5-9	Care and disease manager
60	F	50-55	Nursing Diploma	-	NR, NR	15-19	Case and care manager
61	F	45-49	Nursing Diploma	Community nursing	NR, Hospital and community	10-14	Community Nurse
62	F	45-49	Nursing Diploma	-	1, Hospital and community	25-29	Home care nurse
63	F	45-49	Nursing Diploma	-	NR, NR	20-24	NR
64	F	56-60	Nursing Diploma	-	24, Hospital	5-9	Case, care, and disease manager
65	F	50-55	Nursing Diploma	-	22, Hospital	5-9	Community Nurse
66	F	50-55	Nursing Diploma	-	22, Hospital	5-9	Case and care manager
67	F	45-49	Nursing Diploma	-	NR, NR	25-29	Case and care manager
68	M	40-44	BNS	-	2, NR	10-14	Case manager

Legend: F female; M male; BNS Bachelor of Nursing Science; MNS Master of Nursing Science; FCN Family and Community Nurse; NR Not Reported

Table S4. Facilitating and hindering factors emerged from project work and interviews: levels, themes and quotes

Facilitating factors			Hindering factors	
Level	Themes	Quotes	Themes	Quotes
Nurse	Changing the mind (professional perspective)	We were more performance-oriented at the beginning, now we've become, we've started working more in terms of patient care (I3)	Being focused on self	Because, in my opinion, instead of looking for an overall view we focus on our own, how is it called, only on our own step, you know, so we lack an overview (I13)
	Moving to a user-centered model	It's about switching from an activity which is not only performance-oriented to a much more complex, structured and personalized organizational model, focused on the users' needs (I14)		
	Understanding your own responsibilities	Aware of what can happen, of your responsibilities (I1)	Being focused on nursing tasks	While nurses are overloaded with many things (I11)
	Having time to patient care	Devoting the right amount of time to people and avoiding rushing them (I1)	Lacking in time	Another problem is that in the time dedicated to care a large part of the time goes into it, which is related to bureaucracy, logistics in general (cars, equipment, medicines, materials), keeping agendas, etc., for which other administrative and auxiliary staff can be employed to support according to well-codified methods (PW4)<br>Lacking is time to devote to certain situations, maybe to take over these situations (I6)
	Possessing advanced competencies	The sense of responsibility towards the patient is great and one does not shy away from difficulties but requires continuous updating of technique and science (PW21)<br>Working within a family is becoming more and more complicated and complexity also implies competence, so I think that's also about innovation (I2)	Being unprepared	As for education, prevention, acting in a preventive, proactive manner, maybe in advance, and working on such things, I think these are the aspects I feel weaker (I11)
	Being able to anticipate the care needs	In addition, the aspect of "proactivity" can also be improved, trying to anticipate the needs and requirements, taking responsibility for some choices more decisively (PW43)<br>Now instead this model forces us to work in a proactive way (I2)	Lacking in prioritization skills	I'm in a hurry, I have fifty of them, I need to finish that, I must help my colleague, and I also have to stop and talk to these relatives (I6)
	Being further trained by expert nurses	I'm a long-time member of the group and I keep supporting the two colleagues in my field, the new ones, giving them information, strategies to solve certain problems (I2)		

	Accessing advice from specialized nurses (e.g., palliative care)	Currently, the consultation is provided only by the doctor for people admitted to hospital wards, but at the nursing level it is an activity that is already carried out between colleagues, only not yet formalized (PW15)<br>Now have a nurse who specifically provides those treatments, so we're carrying out this implementation in some areas, to be more specialized (I8)	Lacking expert/specialized nurses in palliative care	It is necessary that the FCNs skills are integrated with two extremely important territorial specialist services, and they are the Palliative Care team and the management of the home pediatric unit (PW13)<br>As for palliative care, in my opinion, in which we're now, as in other cases, short on resources, but there isn't a specific nursing role focused on palliative care (I10)
	Being able to work digitally	Use software and computer applications useful for the assessment and management of the population in charge, using tools for remote monitoring and remote assistance (PW2)<br>And an even more innovative aspect could be the introduction of telemedicine, because it might somehow replace the nursing consultation provided at home through a portal and by organizing consultations, assessments, with relatives, obviously, if they're willing to accept educational or preventive interventions, so it could work from this point of view (I3)		
Micro system	Being close to users, proximal and knowing the users' and family needs	I think it represents about 80% of it. Not only the user's needs but also those of the family supporting them (I9)<br>Closeness, so familiarity, you know, a more empathic kind of relationship with people (I5)	Dealing with different needs perceived by users	The educational aspect in relation to the young population, still quite healthy in preventive terms of lifestyle, is lacking, or present only in a secondary or patchy manner (I10)
	Building a partnership with families and users	The empathic approach must occur with both the patient and the caregiver (PW53)<br>A positive situation at home, a state of wellbeing or the stabilization of the disease are reached and home care turns out to be adequate, it's not only the family who benefit from this, because in small realities, in small communities, there are positive effects on the community as a whole, and this kind of pathway makes it possible for them to have a reference person (I4)	Lacking users' involvement	Very often caregivers do not feel safe to perform therapy because they do not know how to evaluate the patient (PW51)<br>Over the last years we've seen many performances done, and very little desire for involvement, to be honest (I12)
	Knowing/daily experiencing the community	It's an added value because you can use family networks, relatives' networks, they all know each other, also within the community of a town (I9)	Being unfamiliar with the community	The service didn't know the social, cultural and economic context in which the patient lives. (PW9)<br>There also exist situations, contexts where the involvement of a person, of an external worker is almost interpreted as a disturbing element (I12)

	Expanding further knowledge on the community and in its development	The more we are in one area, the better, (…) in this way you have the chance to get to know the community in a broader sense, in a more precise manner (I4)	Lacking in understanding the multidimensionality of the community (multiculturalism)	Taking charge of foreign patients (PW3)<br>Also because of the structure and the complexity of that area, (…) where there are peculiarities and problems also related to the kind of population living in such dimensions, maybe because of the presence of immigrants or small organizations that aren't always available to interface with the rest of the city (I12)
Meso system	Working in a team sharing information	Working in a team presupposes a relationship between people, physical proximity between various professionals helps the relationship, direct vision, eye contact creates an important communicative bond (PW21)<br>We started using the specific home care documentation, (…), led the involvement of the general practitioners, that was truly positive (I6)<br>Working as a community nurse, meaning that my way of acting has always been based on a connection with social workers and general practitioners (I1)<br>A strategy to promote ongoing communication, ongoing information transfer within the group (I2)	Lacking in communication and collaboration	There is the difficulty of interrelationship between general practitioner and social worker (…) perhaps due to work difficulties or poor collaboration in the management of the case (PW8)<br>We are short on doctors, (…), it's more difficult to ensure that kind of collaboration we had before, you know (I6)<br>We are not used to meeting social workers, so to getting in touch with that part which is still external, to creating a network with the community, through meetings (I3)
	Being supported by the nurse manager	Nurse managers are also encouraging us and insisting on this concept, we talk about it, so we tried to see, to do, what do you say, if one chose to try the others usually followed them (I1)	Implementing a new model without any support	Well, nobody supported me, but this is an example (I7)
	Experiencing a positive organizational atmosphere	Positive atmosphere that allows the information transfer within the group, so the ability to transfer the information to everybody, to motivate them also in relation to changes (…) to transfer information you need to build good relationships, and sometimes the atmosphere influences the relationship (I13)		
	Being internally supported by human resources service	The fundamental element is the need to channel, to organize the available human resources to improve the quality of the response offered by the service (I14)	Delivering inconsistent care due to the high turnover	The organization rotates staff a priori after a certain number of months, to avoid having exactly the opposite of what should be the health agreement between citizens and nurses (I4)<br>There's also the organizational part, regarding the resource allocation, because the workload has increased, so care is increasing, (…) we can't think we would be able to do the same job with the same resources, so we should also insist on an organizational kind of aspect, which is certainly associated with integration (I2)

Exo system	Involving general practitioners	But starting from now with the involvement of general practitioners, but only because nowadays doctors still play a very important role within families, within the population (I12)	Working in immature systems	I perceived this, that we're still not mature and ready enough, speaking both of people and services around us, to implement this model in a complete manner (I2)
	Being formally recognized by institutions/policies	The institutions, the local authorities are willing to do this too, because there's been, I don't think it was done by word of mouth, but anyway it was maybe a source of pride for the local authorities to have a nurse withing the community (I9)	Lacking in the expected support	Sometimes we should also be supported instead of being blocked, instead of compensations, of newspapers (I11)
Macro system	Perceiving progressive awareness about the importance of the FCN	There is awareness of the need to promote health oriented towards the empowerment of subjects and contexts, conducted by and with the community (including school), referring to the model of initiative medicine, but professional action is not yet consolidated, in fact there is no reference to the Population Health Promotion Model, the Expanded Chronic Care Model and Population Health Management (PW2)<br>Their recruitment, with the involvement of the nurse, explaining to the families themselves what's the role of the community nurse, this should make the difference, or at least start to make the difference (I12)	Perceiving non-recognition by FCN	In my experience, the role of the FCN is not always recognized (PW5)<br>But I was disappointed because nobody understood the path, I was promoting to educate them on such things as transportation, nobody understood (I7)
			Perceiving different care service expectations by the community	Discrepancy between the expectations of the user/family members and the real perceived situation (PW7)<br>There are also different expectations, you know, then of the district nurse, (…) there are different level on interpretation and consideration of our service (I12)
	Being accepted by the community	As for families, there are positive families who welcome us with enthusiasm, also in relation to preventive, educational and more, intellectual interventions (I3)	Feeling the community unpreparedness regarding the potentialities of the FCN	"Difficulty in accepting the service" (PW7)<br>As for families, (…) don't see the problem and don't feel the need and don't want to work on a prevention level (I3)
	Creating network of services focused on users' needs	We should reorganize the system in order to pay more attention to people, beyond the need for resources, that's it (I10)	Missing collaboration among services	We collaborate with various figures within our company and sometimes outside (see voluntary associations or the Municipality) but there is no real global care, everyone does their own bit but what has always been missing is communication between services (PW22)<br>I noticed that years ago, before covid, we managed to follow people better in their, in everything, in their complexity, I managed to follow them better, the deficiencies are instead communicated occasionally, but once there was more collaboration with the cardiology

				ward, so they called me, they communicated us this, which is not happening anymore, the diabetics (I7)
	Involving the third sector	Basically, paying now a bit more attention, with a more open mind while searching for something to which we usually don't pay that much attention, that is the third sector (I1)	Missing the third sector involvement	Looking for ways to get to know all the voluntary associations present in the area and soon after it can be developed (PW15)<br>The aspect of the case manager is currently not detached from the performance sphere but is devoid of relationships and methods of integration with the third sector in the development. (PW58)<br>There aren't connections with the third sector, sadly, and this is a flaw because it'd be good if we created links, paths, roads facilitating the user's situation in this case (I3)

[3]Legend: I Interview; PW project work; FCN, Family and Community Nurse
